# Why do the Nordic countries allow colposcopy to be a weak link in the cervical cancer screening chain?

**DOI:** 10.1111/aogs.70166

**Published:** 2026-02-17

**Authors:** Ilkka Kalliala, Hanna Milerad, Ameli Ellen Claesdotter Trope, Anne Hammer

**Affiliations:** ^1^ Department of Obstetrics and Gynecology Helsinki University and University Hospital Helsinki Helsinki Finland; ^2^ Medical Unit Gynecology and Reproductive Medicine Karolinska University Hospital Stockholm Sweden; ^3^ Section for Cervical Cancer Screening, Cancer Registry of Norway Majorstuen Norway; ^4^ University Clinic for Gynecological HPV‐Related Disease, Department of Obstetrics and Gynecology, Gødstrup Hospital Herning Denmark; ^5^ Department of Clinical Medicine Aarhus University Aarhus Denmark

Cervical cancer screening has led to a significant decrease in cervical cancer incidence and mortality in the Nordic countries.^1^ The purpose of screening is to identify the few healthy, asymptomatic women who are at increased risk of cervical precancer who need treatment to avoid progression to cancer, and to diagnose cervical cancer at an early stage to reduce mortality. Thus, in screening, we deliberately, although selectively, try to convert ostensibly healthy individuals into “sick,” based on the results of a screening test. By design, population‐based screening prioritizes sensitivity over specificity, and hence, false‐positive results are therefore an inherent and acceptable consequence of strategies aimed at reducing cervical cancer incidence and mortality. This is in contrast with diagnostic testing, which happens when investigations are undertaken due to symptoms presented by the patient, indicating the presence of an underlying cause for the symptoms already before examining the patient. In such a setting, it is rare for a healthy individual to be wrongly diagnosed as sick.

## 1 SCREENING IS A CHAIN: COLPOSCOPY CANNOT BE THE WEAK LINK

The core challenge in screening programs is to optimize the entire screening cascade so that its benefits (prevented cancers, reduced morbidity and mortality) outweigh the harms (anxiety, over‐referral, overdiagnosis, overtreatment, adverse effects of treatment). Consequently, it is important that as many with the disease as possible are detected through screening without increasing the number of false positives.

A sufficient and high‐quality cervical cancer screening program relies not only on the performance of the screening test but also on a chain of interlinked processes: invitation/participation, primary screening test, examination with colposcopy for correct diagnostics, colposcopy‐directed excisional treatment (leading to significantly smaller cone specimens without compromising treatment adequacy),^2^ follow‐up after treatment, and finally, quality assurance and audit of the screening program.

Colposcopy is a pivotal intervention, where cervical precancers are identified and treated before malignant transformation. It is an important step in preventing cancer. Yet, unlike other links in the screening chain—which typically demand certification, standard operating procedures, and external quality assurance—colposcopy often remains permissive. In the Nordic countries any gynecologist is allowed to perform it. No requirements. No certification. No quality assurance. No cervical cancer screening program would allow uncredentialed staff to perform human papillomavirus (HPV) testing in the laboratory, cytological evaluation, issue invitations, or interpret biopsies. This is also the case in the Nordic countries. Then why is colposcopy, the procedure used for diagnostic work‐up of screen‐positive women, permitted to be the weakest link in a system predicated on precision and reliability?

## 2 COLPOSCOPY OPERATES IN A LOW PRETEST PROBABILITY CONTEXT

Even among screen‐positive individuals, the prevalence of clinically significant cervical precancer (HSIL/CIN2+) at colposcopy is modest.^3^ Depending on the HPV genotype detected in the screening sample and the results of, for example, cytology‐based triage, the prevalence of CIN2+ ranges approximately from 10% to 50%.^3^ The positive predictive value (PPV) (i.e., how many of those with a positive test have the outcome) of colposcopy for CIN2+ detection is highly sensitive to both test performance and underlying prevalence of CIN2+.

Sensitivity measures the ability of the screening test to correctly identify and diagnose those with CIN2+. Specificity, on the other hand measures how well the test recognizes those who do not have cervical disease (i.e., healthy). Sensitivity matters because missing true disease at this pivotal point forfeits the primary preventive benefit of screening; specificity matters because it constrains unnecessary biopsies and treatments.

Colposcopy has a reported sensitivity ranging from 30% to >90% and a specificity of 34%–76%.^4^, ^5^ The diagnostic test in question here is colposcopy and both the specificity and sensitivity of colposcopy mainly depend on one thing: the colposcopist. The medical assessment at colposcopy is complex, and will get even more intricate as effective screening and vaccination efforts are launched, leaving solely women with risk factors and/or comorbidities still at risk for cervical cancer. Thus, it is not just about the colposcopist's ability to recognize abnormalities and collect biopsies, but also the ability to integrate knowledge on HPV genotype, cervical cytology result, screening history, previous history of cervical precancer, risk factors such as smoking, comorbidities, HPV vaccination status, and patients' compliance. The colposcopist therefore has a critical role in cervical cancer screening: to correctly recognize and decide whom to treat.

## 3 HOW OPERATOR SKILL CHANGES THE POSITIVE PREDICTIVE VALUE OF COLPOSCOPY

If we assume that the prevalence of CIN2+ among those referred to colposcopy is 10%, an increase in the sensitivity of colposcopy from 30% to 90% would result in almost threefold PPV (from ~4.8% to ~13.2%). If we further improve specificity from 34% to 76% at the same sensitivity of 90%, the PPV would further over double to 29.4%.

The clinical implication is twofold: with a sixfold increase in sensitivity and specificity more cases of true disease would be detected per colposcopy performed, while the ratio of unnecessary biopsies to true disease improves, reducing harm per case detected. This demonstrates that in a low‐prevalence context, modest gains in sensitivity and especially in specificity can yield substantial improvements in net benefit of the colposcopy procedure.

Due to widespread HPV vaccination in the Nordic countries and increasingly effective primary HPV‐based screening, the prevalence of CIN2+ will likely be lower among referrals in the future. Holding test characteristics constant, the PPV declines as prevalence falls. Therefore, without active quality optimization, the downstream yield of colposcopy will diminish and increase the proportional burden of false positives and unnecessary procedures.

## 4 LESSONS FROM OTHER SCREENING PROGRAMS: ACCOUNTABILITY

In colorectal cancer screening, colonoscopy of primary screen‐positive individuals cannot be performed by all gastroenterologists or gastric surgeons: The Nordic countries enforce strict credentialing and minimum annual case numbers for independent practice. Individual‐level quality indicators are mandated and audited, including adenoma detection rate, cecal intubation rate, withdrawal time, and complication rates. Participation in national quality registries is commonplace, with public or institutional reporting and remediation pathways. In breast cancer screening, the radiologists and technologists typically require program‐specific accreditation, and key performance indicators are tracked at reader and unit levels: recall rate, cancer detection rate, interval cancer rates, etc.^6^, ^7^

These examples demonstrate that rigorous credentialing plus continuous quality monitoring can be implemented, sustained, and tied to outcomes. They also show that individual operator metrics drive improvement and protect patients.

## 5 WHY IS COLPOSCOPY NOT ACCOUNTABLE TO THE SAME STANDARDS?

To prevent colposcopy from remaining the weak link in cervical cancer prevention, cervical cancer screening programs should adopt the same rigor for diagnostics and treatment that already govern laboratory testing and diagnostic pathology.

Being allowed to participate in screening and perform colposcopies should also have entrance and ongoing requirements and certification, individual‐level quality metrics and thresholds, mandatory participation in a national or regional colposcopy quality registry with confidential feedback, and tiered remediation, and integration with screening registries.

## 6 WHY ARE WOMEN SCREENED WITHOUT ROBUST QUALITY CONTROL IN COLPOSCOPY?

There is no defensible rationale to exempt colposcopy for diagnostics and treatment from the quality expectations applied to other cervical cancer screening components. Evidence and consensus guidelines for quality control of colposcopy are widespread and endorsed by the European Federation for Colposcopy and the British Society for Cervical Cytology and Pathology. Historical practice patterns, perceived operator scarcity, individual financial interests, and the misconception that colposcopy is a benign, low‐stakes procedure cannot play a role anymore. It is a part of a screening chain and the stakes are high:

1. Missing invasive cervical cancer
2. Unnecessary procedures leading to physical and/or psychological harm
3. Unnecessary burden on and cost for the health care system

As cervical cancer incidence falls, the marginal value of each correctly managed referral increases—and the cost of each error grows in relative terms. The ethical obligation is clear: align colposcopy with the same standards of training, measurement, and accountability that govern the rest of the screening pathway and other cancer screening programs.

Cervical cancer screening succeeds only when every link in the chain is strong. In colposcopy, operator sensitivity and specificity materially determine both patient harm and the overall program efficiency. Other screening domains prove that rigorous credentialing and individual‐level quality monitoring are feasible and effective. Implementing standardized training, individual metrics, and quality registries are not an administrative burden or luxury—they already are a necessary safeguard and quality indicator.

## FUNDING INFORMATION

The authors have nothing to report.

## CONFLICT OF INTEREST STATEMENT

AH has received an honorarium from Exeltis outside the submitted work. The remaining authors declare no conflicts of interest.

## Data Availability

Data sharing not applicable to this article as no datasets were generated or analyzed during the current study.
